# 2286 A CTSA External Reviewer Exchange Consortium: Description and lessons learned

**DOI:** 10.1017/cts.2018.39

**Published:** 2018-11-21

**Authors:** Margaret Schneider, Tanya Mathew, Madeline Gibson, Christine Zeller, Hardeep Ranu, Adam Davidson, Pamela Dillon, Nia Indelicato, Aileen Dinkjian

**Affiliations:** 1 RD and Team Building Administrator, Southern California Clinical and Translational Science Institute, Los Angeles, CA, USA

## Abstract

OBJECTIVES/SPECIFIC AIMS: To share the experience gained and lessons learned from a cross CTSA collaborative effort to improve the review process for Pilot Studies awards by exchanging external reviewers. METHODS/STUDY POPULATION: The CEREC process is managed by a web-based tracking system that enables all participating members to view at any time the status of reviewer invitations. This online tracking system is supplemented by monthly conference calls during which new calls for proposals are announced and best practices are identified. Each CTSA hub customized the CEREC model based on their individual pilot program needs and review process. Some hubs have supplemented their internal reviews by only posting proposals on CEREC that lack reviewers with significant expertise within their institutions. Other hubs have requested 1–3 external reviewers for each of their proposals or a selection of most promising proposals. In anticipation of potential scoring discrepancies, several hubs added a self-assessment of reviewer expertise and confidence at the end of each review. If a proposal is on the cusp of fundability, then the reviewers’ self-assessment may be taken into account. In addition to the tracking data collected by the online system, a survey of CEREC reviewers was conducted using Qualtrics. RESULTS/ANTICIPATED RESULTS: Across the 144 proposals submitted for reviews, CEREC members issued a total of 396 email invitations to potential reviewers. The number of invitations required to yield a reviewer ranged from 1 to 17. A total of 224 invitations were accepted, for a response rate of 56%. An external reviewer was unable to be located for 5 proposals (3%). Ultimately, 196 completed reviews were submitted, for a completion rate of 87%. The most common reasons for non-completion after acceptance of an invitation included reviewer illness and discovery of a conflict of interest. CEREC members found the process extremely useful for locating qualified reviewers who were not in conflict with the proposal being reviewed and for identifying reviewers for proposals related to highly specialized topics. The survey of CEREC reviewers found that they generally found the process easy to navigate and intellectually rewarding. Most would be willing to review additional CEREC proposals in the future. External reviewer comments and scores were generally in agreement with internal reviewer comments and scores. Thus, hubs could factor in external reviewer scores equally to internal reviewer scores, without feeling compelled to calibrate external reviewer scores. Overall, through CEREC external reviewers, mainly due to the stronger matching of scientific expertise and reduction of potential bias, the quality of reviews appear to be higher and more pertinent. DISCUSSION/SIGNIFICANCE OF IMPACT: Some aspects of the process emerged that will be addressed in the future to make the system more efficient. One issue that arose was the burden on the system during multiple simultaneous calls for proposals. Future plans call for harmonizing review cycles to avoid these overlaps. Efficiency also will be improved by optimizing the timing of reviewer invitations to minimize the probability of obtaining more reviews than requested. In addition to the original objective of CEREC, the collaboration has led to additional exchange of information regarding methods and processes related to running the Pilot Funding programs. For example, one site developed a method using REDCap to manage their reviewer database; an innovation that is being shared with the other CEREC partners. Another site has a well-developed process for integrating community reviewers into their review process and is sharing their training materials with the remaining CEREC partners.

